# Comparison of the vaginal microbiota diversity of women with and without human papillomavirus infection: a cross-sectional study

**DOI:** 10.1186/1471-2334-13-271

**Published:** 2013-06-10

**Authors:** Weijiao Gao, Jinlong Weng, Yunong Gao, Xiaochi Chen

**Affiliations:** 1 Key laboratory of Carcinogenesis and Translational Research (Ministry of Education), Department of Gynecologic Oncology, Peking University school of Oncology, Peking University Cancer Hospital and Institute, No 52, Fucheng Road, Haidian District, Beijing 100142, PR China; 2 First Dental Clinic, Peking University School and Hospital of Stomatology, 37 Xishiku Dajie, Xicheng District, Beijing 100034, PR China; 3 Department of Oral Biology, Peking University School and Hospital of Stomatology, No 22, Zhongguancun Nandajie, Haidian District, Beijing 100081, PR China

**Keywords:** Bacterial vaginosis (BV), Human papillomavirus (HPV), Polymerase chain reaction-denaturing gradient gel electrophoresis (DGGE), Vaginal microbiota

## Abstract

**Background:**

The female genital tract is an important bacterial habitat of the human body, and vaginal microbiota plays a crucial role in vaginal health. The alteration of vaginal microbiota affects millions of women annually, and is associated with numerous adverse health outcomes, including human papillomavirus (HPV) infection. However, previous studies have primarily focused on the association between bacterial vaginosis and HPV infection. Little is known about the composition of vaginal microbial communities involved in HPV acquisition. The present study was performed to investigate whether HPV infection was associated with the diversity and composition of vaginal microbiota.

**Methods:**

A total of 70 healthy women (32 HPV-negative and 38 HPV-positive) with normal cervical cytology were enrolled in this study. Culture-independent polymerase chain reaction-denaturing gradient gel electrophoresis was used to measure the diversity and composition of vaginal microbiota of all subjects.

**Results:**

We found significantly greater biological diversity in the vaginal microbiota of HPV-positive women (*p* < 0.001). *Lactobacillus*, including *L. gallinarum*, *L. iners* and *L. gasseri*, was the predominant genus and was detected in all women. No significant difference between HPV-positive and HPV-negative women was found for the frequency of detection of *L. gallinarum* (*p* = 0.775) or *L. iners* (*p* = 0.717), but *L. gasseri* was found at a significantly higher frequency in HPV-positive women (*p* = 0.005). *Gardnerella vaginalis* was also found at a significantly higher frequency in HPV-positive women (*p* = 0.031). Dendrograms revealed that vaginal microbiota from the two groups had different profiles.

**Conclusions:**

Our study is the first systematic evaluation of an association between vaginal microbiota and HPV infection, and we have demonstrated that compared with HPV-negative women, the bacterial diversity of HPV-positive women is more complex and the composition of vaginal microbiota is different.

## Background

Microbes inhabit virtually all sites of the human body and play an important role in human health, yet we know relatively little about them. The Human Microbiome Project (HMP) [1], funded by the National Institutes of Health Roadmap for Biomedical Research, was implemented in 2007. To date, the HMP has released over 100 million 16S rRNA gene reads and more than 8 trillion bytes of shotgun metagenomic sequences [2]. Studies of the gastrointestinal microbiota showed that bacteria maintain homeostasis with the host in a healthy gut [3]. However, when bacterial dysbiosis (microbial imbalance) occurs in the gut, the host may experience inflammation, a loss of barrier function, and possibly serious disease such as ulcerative colitis, Crohn’s disease and colorectal cancer [4-6].

The female genital tract is an important habitat for human microbiota. Investigation of “normal” vaginal microbiota typically reveals *Lactobacillus* species as the predominance genus in the vagina, which helps to promote a healthy vaginal milieu [7]. Bacterial vaginosis (BV), characterized by a loss of indigenous *Lactobacillus* species and a concurrent overgrowth of anaerobic bacteria, has been associated with vaginal discharge syndrome, poor pregnancy outcomes, pelvic inflammatory disease, post-operative wound infections, and endometritis after elective abortions [8-11]. Additionally, BV predisposes women to infection by human papillomavirus (HPV) [12]. Guo et al. [13] reported that compared with women without BV, those with BV had a lower clearance of HPV. And Dols et al. [14] resisted that in women with HPV infection, the prevalence of *L. crispatus* was significantly reduced and there was a shift in the composition of the *Lactobacillus* microbiota in HPV infection.

Persistent HPV infection is the central factor in the development of cervical cancer, and is a prerequisite for progression to high-grade cervical lesions [15]. However, few HPV infections progress to cervical cancer, and most HPV infections are eventually cleared [16]. The reason(s) why high risk HPV infection is cancerous in some women but not others is unknown. Some studies imply that BV is associated with HPV acquisition; a meta-analysis of twelve studies with a total of 6,372 women indicated a positive association between BV and HPV infection, with an overall estimated odds ratio of 1.43 (95% confidence interval, 1.11–1.84) [12]. However, these studies focused on the relationship between BV (diagnosed using clinical Amsel criteria or Nugent’s score) and HPV infection [17,18], and did not examine the composition of vaginal microbial communities involved in HPV acquisition.

It is difficult to assess the microbial community in an environment where more than 80% of microbiota is nonculturable (such as the vagina) [19,20]. However, the advances of molecular biotechnology, such as culture-independent polymerase chain reaction-denaturing gradient gel electrophoresis (PCR-DGGE), enable better characterization of complex microbial communities [21,22]. The combination of PCR and DGGE allows for the rapid and reliable examination of the vaginal microbiota, and these techniques have been used widely in bacterial microbiota studies [23-25]. The method allows numerous samples to be screened, because the microbial nucleic can be derived directly from human specimens, without the need for culture enrichment. In addition, PCR-generated DNA fragments of the same length but different base-pair sequences can be separated by DGGE. Although PCR-DGGE has some shortcomings, including the fact that it needs costly equipment, the “GC clamp” is very expensive, and the formamide is poisonous, it is a powerful tool for microbiota studies. But the next generation sequencing [26] might will replace the current molecular biotechnology in the near future.

To examine the relationship between the diversity and composition of vaginal microbiota and HPV positivity, we used PCR-DGGE to examine the vaginal microbiota of 38 HPV-negative and 32 HPV-positive healthy women with no BV (using Amsel criteria). To our knowledge, this is the first study of the association between HPV infection and vaginal microbiota to use a molecular biological technique to examine the microbiota.

## Methods

### Subject selection

A total of 100 healthy women (50 HPV-negative and 50 HPV-positive) with normal cervical cytology, who accepted a routine gynecological examination and Thinprep Cytology Test (TCT) in Beijing Cancer Hospital from January 2012 to June 2012, were initially recruited for this study. Subsequently, TCTs were reexamined by two cytologists and HPV infection was reexamined by PCR. If there was a difference between the first and second TCT reports, or between the results from the two cytologists performing the second TCT, the woman was excluded from the study. In addition, if a recruited woman was infected with more than one HPV type, or there was a difference between the initial HPV report using Hybrid Capture II and the second HPV report using PCR, the woman was excluded from the study. A total of 30 women were excluded from the study based on these four criteria. Inclusion criteria were age <50, no BV by the Amsel method, no use of antibiotics or vaginal antimicrobials (orally or by topical application in vulvar/vaginal area) in the previous month, and no vaginal intercourse or vaginal lavage within the last 3 days. All subjects were free of systemic diseases such as diabetes, autoimmune disease, and malignant tumors. Informed written consent was obtained from all participants prior to enrollment. This study was approved by the ethical committee of Peking University Cancer Hospital and Institute, Beijing, China.

### Sample collection and preparation

When women underwent genital examination, a sterile swab sample was taken from near the vaginal fornix and cervix from each participant. The swab was placed into 1 ml sterile saline, placed on ice packs immediately, and transferred to the laboratory within 30 min. The sample was pelleted by centrifugation at ≥10,000 × g (25°C) for 10 min and stored at −80°C until further analysis, as previously published [27].

### Total bacterial genomic DNA extraction

Bacterial DNA was extracted according to the procedures described by Signoretto [28] and Zijnge [29]. Briefly, the sample was incubated for 1 h at 58°C with 1 ml of lysis buffer (10% SDS and 0.2 mg/ml proteinase K in 25 mM Tris–HCl, pH 8). The sample was then incubated at 80°C for 10 min to inactivate the proteinase K. DNA was purified from the lysate by repeated phenol-chloroform-isoamyl alcohol extraction, precipitated with sodium acetate and ethanol, dissolved in 100 μl sterile Milli-Q water and stored at −20°C in aliquots. The concentration of extracted DNA was determined by a Nano Photometer™ Pearl ultramicro ultraviolet spectrophotometer (Implen, Munich, Germany). The quality of the DNA was checked by agarose gel electrophoresis. All DNA was stored at −20°C before further analysis.

### Nested PCR

The hypervariable region chosen for amplification can influence the PCR-DGGE profiles. The V2–V3 region of the 16S rRNA gene is reported to be the most reliable [30,31]. The consensus primers used in this study were as follows: S-D-Bact −0008-a-S-20/ S-*-Univ-1492- b-A-21: AGA GTT TGA TCC TGG CTC AG/ ACG GCT ACC TTG TTA CGA CTT; and HDA1- GC/ HDA2: CGC CCG GGG CGC GCC CCG GGC GGG GCG GGG GCA CGG GGG GAC TCC TAC GGG AGG CAG CAG/ GTA TTA CCG CGG CTG CTG GCA. The primers used in this study have been published previously [32,33].

The first PCR mixture contained 100 ng of DNA template, 5 pmol of each primer, 25 μl of 2× PCR Master Mix (0.05 unit/μl Taq DNA Polymerase, 4 mM MgCl_2_, 0.4 mM dATP, 0.4 mM dCTP, 0.4 mM dGTP, 0.4 mM dATP and 0.4 mM dTTP) (Fermentas, Vilnius, Lithuania) and RNase-free H_2_O in a final volume of 50 μl. The cycling parameters were 95°C for 5 min; 25 cycles of 95°C for 2 min, 42°C for 30 s, 72°C for 4 min; and a final cycle of 72°C for 20 min. The temperature was held at 4°C following the final cycle [34].

The second PCR mixture contained 5 μl of PCR product from the above reaction, 10 pmol of each primer, 25 μl of 2× PCR Master Mix (0.05 unit/μl Taq DNA Polymerase, 4 mM MgCl_2_, 0.4 mM dATP, 0.4 mM dCTP, 0.4 mM dGTP, 0.4 mM dATP and 0.4 mM dTTP) and RNase free H_2_O in a final volume of 50 μl. The cycling parameters were 95°C for 5 min; 30 cycles of 95°C for 30 s, 56°C for 30 s, 72°C for 1 min; and a final cycle of 72°C for 8 min. The temperature was held at 4°C following the final cycle [35].

### DGGE

The denaturing gradient gel was formed with 8% polyacrylamide stock solution containing either low (40%) or high (70%) concentrations of urea and formamide that increased in the direction of electrophoresis.

PCR products were mixed with a loading buffer containing bromophenol blue, xylene cyanol, and 70% glycerol in TAE buffer (0.02 M Tris base, 0.01 M acetic acid, 1 mM EDTA, pH 7.5) and loaded into the gels. About 20 μl of the reference mixture was combined with loading buffer, and the mixture was loaded so that it flanked the vaginal samples. Electrophoresis was performed for 16 h at 60 V and 58°C. The gels were stained with SYBR Green I (1:10,000) for 30 min.

DGGE images were digitally captured and recorded by the BIO-RAD Gel Doc™ XR^+^Gel Imaging System (Bio-Rad, Hercules, USA) and analyzed by Gel Compare® (Applied Maths, Kortrijk, Belgium). Each gel was normalized according to a DGGE standard marker (10 markers according to the GC base-pair percentage).

### Cloning of excised DGGE bands

The main DGGE bands were excised, and the DNA fragments were amplified with primers HDA1 (without the CG clamp) and HDA2. Amplified material was cloned into the pGM®-T vector using the pGM®-T cloning Kit (Tiangen, Beijing, China), according to the manufacturer’s instructions. The vector was then cloned into *Escherichia coli* Top10, and the extracted plasmid was sequenced by the Beijing Genomics Institute. Sequence identification of the plasmid was performed by searching the NCBI BLAST database.

### Detection of HPV

HPV DNA was extracted from the remnant TCT sample by TIANamp Virus DNA/RNA Kit (Tiangen) according to the manufacturer’s instructions. HPV DNA was first amplified using the HPV L1 consensus MY09/MY11 primer pair, followed by nested PCR with GP5+/GP6+ primers. The PCR amplification was performed as described previously [36,37].

PCR products were subjected to direct DNA sequencing by the Beijing Genomics Institute. The obtained sequences were compared with documented virus sequences available in the GenBank database using the BLAST program. The subjects whose results were different to the previous results from Hybrid Capture or who were infected with multiple HPV genotypes were eliminated from this study. HPV-negative women and women infected with one HPV (single high-risk) subtype were included in this study. The HPV types are not listed in this paper.

### Statistical analysis

The similarities of PCR-DGGE DNA profiles were analyzed with Gel Compare® software (Applied Maths) using Dice’s similarity coefficient. The clustering algorithm, unweighted pair-group method with arithmetic means (UPGMA), was used to calculate the dendrogram.

Vaginal microbiota diversity was expressed by the Shannon–Weiner diversity index [38-40] and calculated using the following formula: *H’* = *−*Σ*p*_*i*_ln*p*_*i*_. For morphological analysis, *p*_*i*_ is the proportion of individuals in the *i*th taxon. For PCR-DGGE analysis, *p*_*i*_ is the importance probability of the bands in a gel lane and measured as *p*_*i*_ = *n*_*i*_*/N*, where *n*_*i*_ is the intensity of a band and *N* is the sum of all band intensities in the densitometry profile. The Mann–Whitney U test and Kruskal-Wallis tests were performed to compare the diversity indices, and *p* < 0.05 was interpreted to be statistically significant.

## Results

### DGGE profiles in HPV-positive and HPV-negative women

A total of 38 HPV-negative women (mean age 37.0) and 32 HPV-positive women (mean age 37.8) were enrolled in this study. There was no significant difference in age between the two groups (*p* = 0.57).

**Figure 1:**
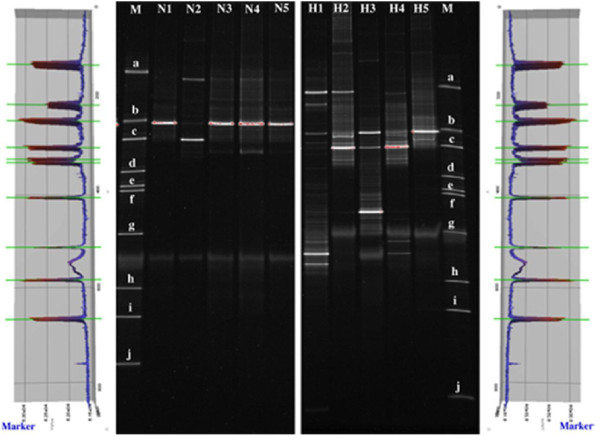
**PCR-DGGE profiles of the predominant bacterial communities in vaginal swabs from five HPV-negative women (left: N1–N5) and five HPV-positive women (right: H1–H5).** Lane M is a marker constructed in this study with the identified bands to facilitate the interpretation of the figure. Bands: **a**: *Lactobacillus fabifermentans* strain, LMG 24284 16S ribosomal RNA; **b**: *Gemella haemolysans* strain, ATCC 10379 16S ribosomal RNA; **c**: *Staphylococcus warneri* strain, AW 25 16S ribosomal RNA; **d**: *Streptococcus mutans* strain, ATCC 25175 16S ribosomal RNA; **e**: *Streptococcus sobrinus* strain, ATCC 33478 16S ribosomal RNA; **f**: *Escherichia fergusonii*, ATCC 35469 16S ribosomal RNA; **g**: *Actinomyces graevenitzii* strain, CCUG 27294 16S ribosomal RNA; **h**: *Actinomyces turicensis* strain, APL10 16S ribosomal RNA; **i**: *Actinomyces viscosus* strain, NCTC 10951 16S ribosomal RNA; **j**: *Actinomyces israelii* strain, CIP 103259 16S ribosomal RNA. The bilateral part of the figure shows the marker files from different DGGE gels. Each peak in the files stands for one marker. A pairwise analysis, using Gel Compare® software, of the gel marker in each gel indicated that the marginal discrepancies of markers between each gel were negligible.

The DGGE profiles of vaginal samples were obtained from all 70 women. Figure 1 shows representative DGGE profiles of vaginal microbiota from the two groups. The left panel depicts DGGE profiles from five HPV-negative women (N1–N5) and the right panel shows profiles from five HPV-positive women (H1–H5). Lane M comprises 10 markers (a–j), and the bands of each lane in each DGGE profile are classified by each marker’s GC base-pair percentage. The bilateral part of the figure shows the marker files from different DGGE gels. Each gel was normalized according to a DGGE standard marker, and each peak in the marker files stands for one marker. Pairwise comparisons between each gel marker using Gel Compare® software demonstrated that the marginal discrepancies of marker bands between each gel are negligible.

The number of bands in the DGGE profile from each woman is shown in Table 1. Previous studies have reported that healthy vaginal microbiota only contains one or two predominant species [41,42]. We compared the number of bands in samples from HPV-positive women versus HPV-negative women. Half of the vaginal samples from HPV-negative women contained more than two bands in their DGGE profiles, whereas 87.5% of HPV-positive women had more than two bands. The mean band number from HPV-negative women was 3.45, and the mean number from HPV-positive women was 6.47 (Chi-squared test, *p* = 0.001).

**Table 1:** Comparison of number of bands between HPV-negative women and HPV-positive women

	**Case number (n and percentage)**			*P *value^*^
	**HPV-negative**	**HPV-positive**	**Total**	
0-2 bands	19 (50%)	4 (12.5%)	23 (32.9%)	0.001
over 2 bands	19 (50%)	28 (87.5%)	47 (67.1%)	

* The Chi-squared test was used to compare the proportion of samples in each category for HPV-negative women versus HPV-positive women. *p* < 0.05 was considered statistically significant.

**Figure 2:**
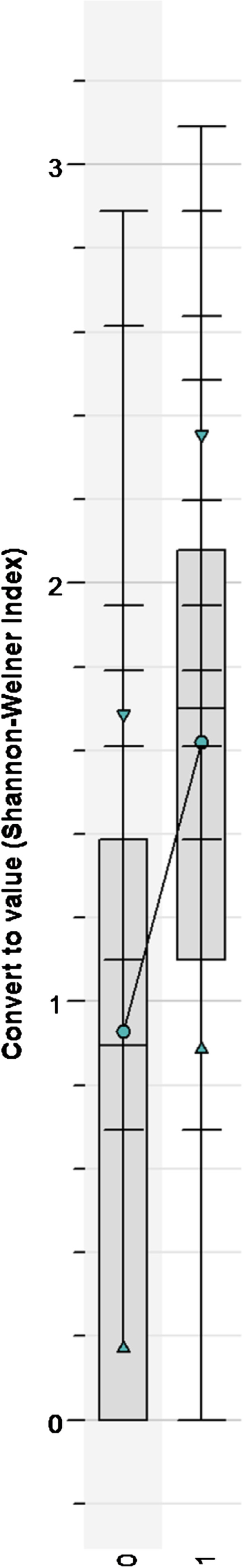
Mann–Whitney U test of the Shannon-Weiner diversity indices from HPV-negative women and HPV-positive women.

Vaginal microbiota diversity was expressed using the Shannon-Weiner diversity index and compared using the Mann–Whitney U test. The diversity index for subjects with and without HPV infection is displayed in Figure 2. We found significantly greater biological diversity in HPV-positive women (mean = 1.64; range, 0 to 3.09) than in HPV-negative women (mean = 0.93; range, 0 to 2.62) (*p* < 0.001).

### Cluster analysis based on DGGE profiles

**Figure 3:**
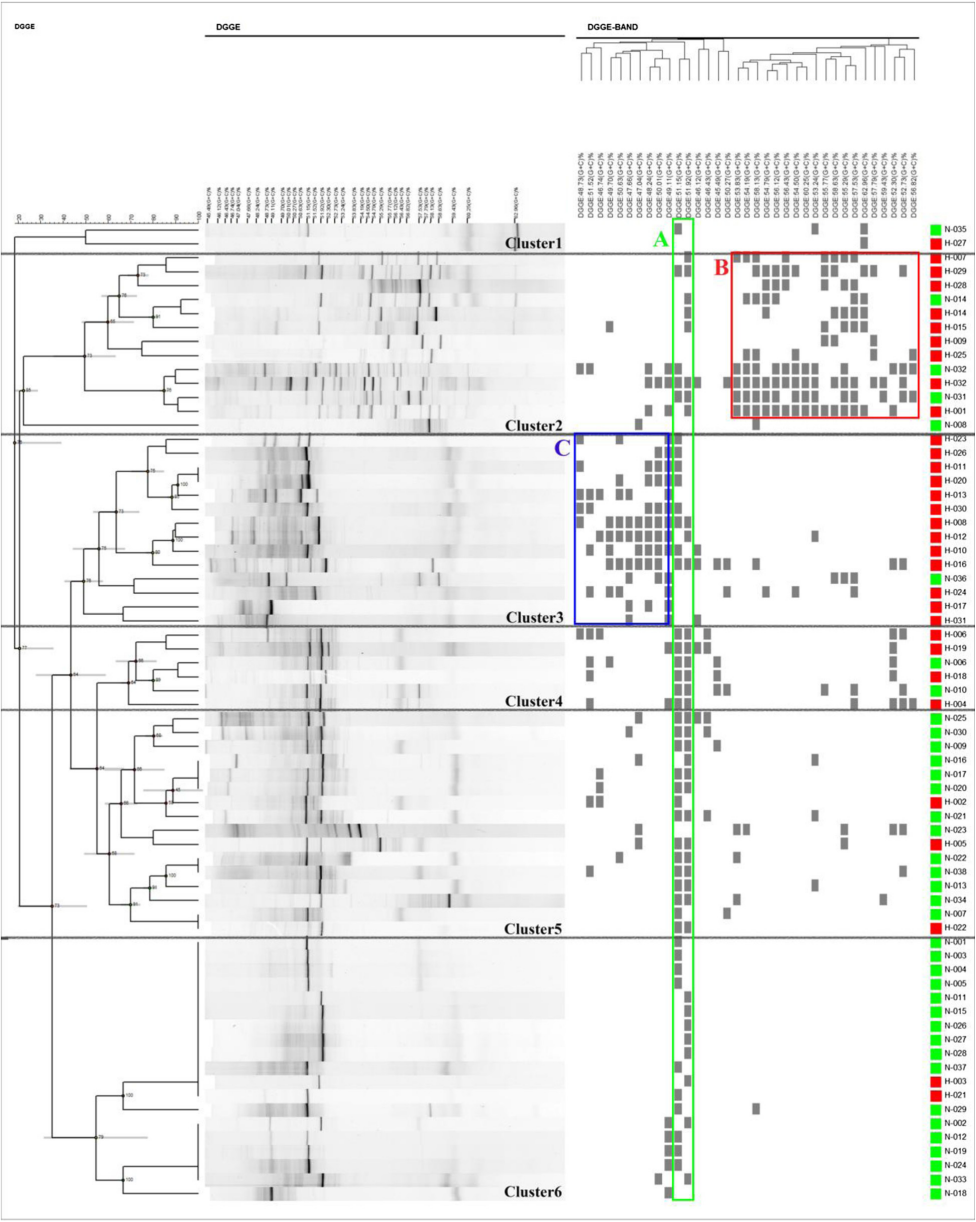
**Hierarchical cluster analysis and discriminative characters analysis of all DGGE profiles.** HPV-negative women are green and HPV-positive women are red. The box around bands **A** is green, the box around bands **B** is red, and the box around bands **C** is blue.

The UPGMA clustering algorithm was used to construct a dendrogram of the DGGE profiles of vaginal microbiota from all 70 women enrolled in this study. Hierarchical cluster analysis of the dendrogram indicated that six clusters were formed, and revealed differences in the composition of vaginal microbiota between HPV-negative women and HPV-positive women (Figure 3). Most samples from HPV-positive women fell within clusters 1–4. Conversely, clusters 5 and 6 primarily comprised samples from HPV-negative women. There were significantly more bands in cluster 1–4 than in clusters 5 and 6, indicating that the vaginal microbiota of HPV-positive women had a greater biological diversity than that of HPV-negative women. Of note, in cluster 6 the DGGE profiles consisted of only one or two bands.

The discriminative character analysis of DGGE profiles showed that the bands in box A are present in both HPV-positive and HPV-negative women (Figure 3). However, the bands in box B mostly appeared in cluster 2 (which primarily consists of HPV-positive women). The bands in box C were detected in cluster 2, cluster 3, and other clusters, but the bands in box C are mostly from HPV-positive women.

We compared the number of bands, Shannon–Weiner diversity index and the proportion of samples from HPV-positive and HPV-negative women in each of the six clusters (Table 2). There were large differences in all three characteristics (all *p* values <0.001). The Kruskal-Wallis test was used to compare the Shannon-Weiner diversity indices from each of the six clusters, and we found that the diversity of at least one of the clusters was significantly different from the other clusters (*p <* 0.001).

**Table 2:** Comparison of the number of bands, Shannon–Weiner diversity index and the proportion of samples in each cluster

		**Cluster 1**	**Cluster 2**	**Cluster 3**	**Cluster 4**	**Cluster 5**	**Cluster 6**	***P*value**
Number of bands		2.00 ± 1.414	9.69 ± 6.223	6.36 ± 3.054	6.17 ± 1.329	4.13 ± 2.187	1.32 ± 0.478	<0.001*
Shannon–Weiner index		0.55 ± 0.777	2.07 ± 0.717	1.75 ± 0.469	1.80 ± 0.214	1.23 ± 0.293	0.22 ± 0.331	<0.001^#^
Group	HPV-negative	1 (50.0%)	4 (30.8%)	1 (7.1%)	2 (33.3%)	13 (81.3%)	17 (89.5%)	
	HPV-positive	1 (50.0%)	9 (69.2%)	13 (92.9%)	4 (66.7%)	3 (18.7%)	2 (10.5%)	<0.001*
	Total	2 (2.9%)	13 (18.6%)	14 (20.0%)	6 (8.6%)	16 (22.9%)	19 (27.1%)	

*The Chi-squared test was used to compare the proportion of samples in each category for HPV-negative versus HPV-positive women. *p* < 0.05 was considered statistically significant.

^#^ The Kruskal-Wallis test was performed to compare the diversity indices, and *p* < 0.05 was interpreted to be statistically significant.

### Identification of vaginal microorganisms

Excised DGGE gel bands were sequenced and BLAST database searches were used to identify 18 bacterial species present in vaginal samples from the 70 women studied. The name of each microorganism, sequence identities and NCBI Sequence ID are shown in Table 3. The sequence identities of most microorganism were above 97%.

**Table 3:** Microorganisms identified in the vaginal tract of all 70 women

**Name of microorganism**	**Identity%**	**Sequence length**	**Genbank accession numbers**
*Aeromicrobium spp.*	92	154	----
*Alloscardovia omnicolens*	99	152	NR_042583.1
*Atopobium vaginae*	97	151	NR_029349.1
*Bifidobacterium scardovii*	98	152	NR_025452.1
*Escherichia fergusonii*	99	172	NR_027549.1
*Finegoldia magna*	99	148	NR_041935.1
*Gardnerella vaginalis*	99	154	NR_044694.1
Lactobacillus gallinarum*	99	174	NR_042111.1
*Lactobacillus gasseri*	99	172	NR_041920.1
*Lactobacillus iners*	98	168	NR_036982.1
*Nocardia spp.*	90	153	----
*Peptostreptococcaceae bacterium*	99	146	NR_041586.1
*Prevotella spp.*	96	163	----
*Pseudobutyrivibrio spp.*	96	146	----
*Streptococcus agalactiae*	99	168	NR_040821.1
*Streptococcus australis*	98	173	NR_036936.1
*Streptococcus intermedius*	99	171	NR_028736.1
*Ureaplasma parvum serovar 3 str.*	99	169	NR_027532.1

*The result of a BLAST search indicated that the similarity score of *L. gallinarum* was same as other *Lactobacillus spp.*, such as *L. crispatus, L. acidophilus, L. amylovorus, L. rhamnosus, L. fermentum, L. helveticus*, *L. kitasatonis and L. ultunensisc.*

*Lactobacillus* was the most predominant genus, and was detected in all women included in this study. The *Lactobacillus* members are grouped into three species: *L. gallinarum* was the most abundant (45/70, 64%), followed by *L. iners* (41/70, 59%) and *L. gasseri* (23/70, 33%) (Table 4). The next most predominant genus was *Gardnerella vaginalis* (14/70, 20%), followed by *Atopobium vaginae* (7/70, 10%). A Chi-squared analysis revealed that there is no difference in the frequency of identification of *L. gallinarum* between HPV-negative and HPV-positive women (*p* = 0.775). Likewise, no significant difference existed in the frequency of identification of *L. iners* (*p* = 0.717) or *Atopobium vaginae* (*p* = 0.150). However, *L. gasseri* and *Gardnerella vaginalis* were isolated more frequently in HPV-positive women than in HPV-negative women (*p* = 0.005 and *p* = 0.031, respectively).

**Table 4:** Number of times the main genus was identified in HPV-negative women and HPV-positive women

**Name of microorganism**	**Times identified in women**		*P *value^*^
	**HPV-negative (n = 38)**	**HPV-positive (n = 32)**	
*Lactobacillus gallinarum*	25	20	0.775
*Lactobacillus iners*	23	18	0.717
*Lactobacillus gasseri*	7	16	0.005
*Gardnerella vaginalis*	4	10	0.031
*Atopobium vaginae*	2	5	0.150

^*^ The Chi-squared test was used to compare the times of the main genus identified in HPV-negative women and HPV-positive women. *p* <0.05 was considered statistically significant.

*L. gallinarum* and *L. iners* are the two bands in box A of Figure 3. *Gardnerella vaginalis* is the predominant genus of the bands in box B, and other bands in box B include *Escherichia fergusonii, Atopobium vaginae, Streptococcus australis, Streptococcus intermedius* and *Alloscardovia omnicolens*. *L. gasseri* is the predominant genus of the bands in box C, and another band in box C is *ureaplasma parvum serovar 3 str*.

## Discussion

Biologic susceptibility to HPV acquisition and immune competence for clearance of an HPV infection can be affected by vaginal bacterial infection, which disrupts the balance of vaginal microbiota [12,43]. Previous studies have not investigated the difference in microbiota communities between HPV-negative and HPV-positive women. To our knowledge, the current study is the first systematic evaluation of an association between vaginal microbiota and HPV infection. Our study analyzes the diversity and abundance of the vaginal microbiota by PCR-DGGE, and compares the microbiota by HPV infection status. This study may be helpful to reveal the role of the vaginal microbiota in the natural history of HPV infection.

If the target 16S rRNA gene copy number in the sample is low, it is likely that it cannot be amplified sufficiently to be visualized as a band. Therefore, the bands generated in DGGE reflect the most abundant genus from each vaginal sample [44]. In our study, the number of DGGE bands in HPV-positive women was significantly higher than that in HPV-negative women. Therefore, we conclude that the vaginal microbiota of HPV-infected women is more complex than that of HPV-negative women in our study.

The Shannon-Weiner diversity index uses the total number and relative intensities of DGGE bands, which makes it more suitable to estimate bacterial diversity than the number of bands alone [45]. A Mann–Whitney U test comparing the Shannon-Weiner diversity index between HPV-positive and HPV-negative women demonstrated that there was significantly greater biological diversity in HPV-positive women than HPV-negative women (*p* <0.001). Other studies have also demonstrated a difference in the abundance of bacterial species present in the vagina when comparing healthy women to those with BV or cervicitis [25,46]. This suggests that an increase in bacterial diversity may be associated with the shift from health to disease. Of note, the opposite is true for oral health, where a decrease in bacterial diversity is associated with disease [47].

The UPGMA clustering algorithm has been used previously [48], and in our study it was used to identify samples that generate similar DGGE profiles. The UPGMA dendrogram showed that six distinct clusters are formed, and that samples from HPV-positive and HPV-negative women tend to be in distinct clusters. The discriminative characters analysis of DGGE profiles indicate that the bands of box B and box C, which are different in clusters 1–4 versus cluster 5–6, might generate the distinction of clusters.

Over 120 species of *Lactobacillus* have been identified, and more than 20 species have been detected in the vagina. However, vaginal microbiota is not reported to contain very many different species of *Lactobacillus*[49]. Typically, one or two *Lactobacilli* species are predominant. For example, *L. crispatus* and *L. jensenii* were the most common genera for white women [50], while *L. crispatus* and *L. gasseri* were more common in Japanese women [51]. Recently, a study from China reported that *L. crispatus, L. iners* and *L. gasseri* were the most common genera in Chinese women [52].

In our study, sequence analysis identified three *Lactobacillus* members: *L. gallinarum*, *L. iners* and *L. gasseri*, all of which are obligately homofermentative species. *L. gallinarum* and *L. iners* are the two bands in box A (Figure 3), and there was no difference between HPV-negative women and HPV-positive women, there was no difference among each cluster. However, *L. gasseri* is the main species in box C, and was detected more frequently in the HPV-positive women (*p* = 0.005). Cherpes et al. reported that *L. gasseri* was vaginal colonization from the rectum [53], and a study of homosexual women found an association between *L. gasseri* and BV [54]. And Dols et al. [14] resisted that the prevalence of *L. crispatus* was significantly reduced and there was a shift in the composition of the *Lactobacillus* microbiota in women with HPV infection.

But we were not able to distinguish *L. gallinarum* from the other *Lactobacillus spp.*, because the BLAST result indicated that the similarity score of *L. gallinarum* was the same as that of other *Lactobacillus spp.,* such as *L. crispatus, L. acidophilus, L. amylovorus, L. rhamnosus, L. fermentum, L. helveticus*, *L. kitasatonis and L. ultunensis*. One possible explanation for this is that the genera of *Lactobacillus spp.* mentioned above have the same DNA sequence as *L. gallinarum* in the V2–V3 region of 16S rDNA. Future research will seek to resolve this issue.

The bands in box B are the primary bands that distinguish cluster 2 from other clusters. The main microorganisms in box B are *Gardnerella vaginalis* and *Atopobium vaginae*. *Gardnerella vaginalis* is a facultative anaerobic bacterium of the *Bifidobacteriaceae* family [55], while the genus *Atopobium* lies within the family *Coriobacteriaceae* and forms a distinct branch within the phylum *Actinomycetes*[56]. Because *Gardnerella vaginalis* and *Atopobium vaginae* were frequently detected in association with BV, Menard deemed the combination of the two bacterial genera as predictive criteria for the diagnosis of BV [57]. In our study, *Gardnerella vaginalis* (10/32 vs. 4/38, *p* = 0.031) and *Atopobium vaginae* (2/38 vs. 5/32, *p* = 0.150) were more frequently detected in HPV-infected women than in HPV-negative women, though the difference of *Atopobium vaginae* in the two groups was not statistically significant.

Because this was a cross-sectional study, we were unable to determine whether a change in vaginal microbiota preceded HPV infection, or whether HPV infection preceded a change in vaginal microbiota. Furthermore, we initially recruited 100 women with normal cervical cytology and excluded 30 from the study, which led to a relatively small sample size. However, because this is the first study to use PCR-DGGE to examine the association between vaginal microbiota and HPV infection, even with the limitations mentioned above we believe it has substantial importance.

## Conclusion

In conclusion, we found that the bacterial diversity and composition in HPV-positive women were more complex than in HPV-negative women. *Gardnerella vaginalis* and *L. gasseri* were detected significantly more frequently in HPV-positive women. Abnormal vaginal microbiota might act as a co-factor for the acquisition of HPV. Continued exploration of the interplay between vaginal bacterial communities and HPV may shed light on biologic susceptibility to HPV infection, as well as immune competence for clearance of HPV, which may provide new insights into the early steps in the development of cervical cancer.

## Abbreviations

BV: Bacterial Vaginosis; DGGE: Denaturing Gradient Gel Electrophoresis; HPV: Human Papillomavirus; PCR: Polymerase Chain Reaction; HMP: the Human Microbiome Project; TCT: Thinprep Cytology Test; UPGMA: Unweighted Pair-Group Method with Arithmetic Means.

## Competing interests

The authors declare that they have no competing interests.

## Authors’ contributions

WJ Gao conducted the PCR-DGGE studies, performed the statistical analysis, participated in clinical sample collection and drafted the manuscript. JL Weng conducted the cloning of DNA fragments and participated in the statistical analysis. YN Gao and XC Chen conceived the study, participated in its design and coordination and helped to draft the manuscript. YN Gao carried out clinical sample collection and was responsible for clinical data. XC Chen helped conduct the molecular biological experiments and was responsible for the experimental data. All authors read and approved the final manuscript.

## Pre-publication history

The pre-publication history for this paper can be accessed here:

http://www.biomedcentral.com/1471-2334/13/271/prepub
